# Why Do Some Ethiopian Women Give Birth at Home after Receiving Antenatal Care? Phenomenological Study

**DOI:** 10.1155/2018/3249786

**Published:** 2018-07-18

**Authors:** Yohannes Mehretie Adinew, Netsanet Abera Assefa, Yimenu Mehretie Adinew

**Affiliations:** ^1^College of Health Sciences and Medicine, Wolaita Sodo University, Sodo, Ethiopia; ^2^College of Public Health and Medical Sciences, Jimma University, Jimma, Ethiopia

## Abstract

**Background:**

In Ethiopia, majority (62%) of pregnant women receive at least one antenatal follow-up, yet only 26% give birth in health facility. Understanding factors underlying this high uptake of antenatal care and low institutional delivery service is critical. Women had antenatal care follow-up means; by default they have access to health facilities. Thus, why do some give birth at home even after receiving antenatal care?* Methods*. Fourteen key informant interviews and six focused group discussions were held among purposively selected women who gave birth in the last 12 months without skilled attendance after receiving antenatal care. The study explored women's perspectives on maternity care, care providers, and factors that influence place of delivery. Interpretative phenomenological analysis was used to examine various behaviors and beliefs of respondents.

**Results:**

Study participants described range of experiences and beliefs that made them give birth at home after receiving antenatal care at health facilities. Four themes emerged from women's description: poor counseling during antenatal care service, traditions, early pregnancy symptoms, and lack of planning in advance for childbirth.

**Conclusion:**

Poor counseling during antenatal care is deterring women from seeking skilled attendance at birth. Thus, healthcare providers need to stress necessity of facility based delivery care during antenatal follow-up counseling.

## 1. Background

Every year, an estimated 300,000 maternal deaths occur worldwide and 99% of them occur in developing countries. Skilled attendance at birth is proved to be “the single most effective intervention in preventing maternal death” [1]. Antenatal care (ANC) provides an opportunity to promote skilled attendance, prevent complications where possible, and ensure that complications are detected and treated early [2, 3].

Childbirth is a vital event in women's life and represents a time of intense vulnerability. Especially in developing countries like Ethiopia where institutional delivery is low [4, 5] complications related to pregnancy and childbirth are leading causes of morbidity and mortality for women of child bearing age [5, 6]. Ethiopia has maternal mortality ratio of 412 per 100,000 live births and home delivery is indicated as the main reason for high maternal death among rural women [7, 8]. Therefore, improved access to emergency obstetric care can reduce this tragic loss of life and vitality up to 75% [9, 10].

Ethiopian healthcare delivery system is classified as primary, secondary, and tertiary levels.* Kebele*, the lowest administrative unit, has at least one primary healthcare unit (health post) where ANC is mostly provided, while Woreda (district) comprises several* kebeles* and is expected to have at least six primary healthcare units (five health posts and one health center) [11].

Despite the continuous effort to encourage institutional delivery, many Ethiopian women still give birth at home. For instance, access to health service has been greatly improved and 94.0% of the population has accessed primary healthcare services [12] and maternal services had been made free but skilled attendance at birth is still very low, 28%, while facility based childbirth is only 26% [5]. Recent studies also suggested that improving access is not sufficient enough to increase use [13].

Though current figures of skilled birth attendance are encouraging, they are not consistent with ANC take-up. ANC coverage (at least one visit) increased from 42.6% in 2011 to 62% in 2016, while percentage of deliveries attended by skilled health personnel increased from 10% to 28% during the same period [5, 14]. As a result, improving maternal health remains a daunting challenge to the nation [15, 16].

A woman who had ANC follow-up is expected to have access to health facilities which may smooth facility based childbirth. However many women in Ethiopia gave birth out of health facilities, either unattended or attended by traditional birth attendants (TBAs), if any, at home despite their ANC follow-up. Thus, this study was aimed at exploring why some women still give birth at home after receiving ANC by analyzing their explanations of preferences in place of delivery and understanding how they perceive, interpret, and weigh factors that influence their place of delivery.

## 2. Methods

### 2.1. Study Design and Area

An exploratory qualitative study was employed. In-depth interviews and focused group discussions were held with purposively selected study participants. Study was conducted from March to June 2016 in two purposively selected districts of Hadiya Zone, Lemo and Gombora, south Ethiopia. Hadiya zone had one hospital, 60 rural health centers (HCs), and 280 health posts (HPs) that serve nearly 1.2 million populations. The zone had eleven districts and the vast majority of the people were rural dwellers. Lemo and Gombora districts had 33,176 and 26,330 individuals of childbearing women, respectively. 33 HPs and 7 HCs were found in Lemo whereas Gombora had 23 HPs and 6 HCs. Each rural kebele in both districts had one HP [17, 18].

### 2.2. Participants

Study participants were women who gave birth in the last twelve months at home, without skilled attendance after receiving at least one ANC follow-up. Diverse participants were recruited to get good representation (monogamous and polygamous, young and old, uneducated and educated) using purposive sampling. Health extension workers of the district identified potential participants.

### 2.3. Sampling Technique and Procedure

Two* kebeles* were selected from each Woreda. Fourteen IDIs and six FGDs were conducted. Each FGD had 8-10 participants. A total of 68 women took part in the study. Information saturation (when ideas started to be repeated and no more new ideas emerged) was used to determine number of IDIs and FGDs.

### 2.4. Inclusion Criteria

Women who had received only ANC service for their most recent childbirth but no facility based delivery care within the last twelve months were included.

### 2.5. Data Collection Tools and Procedures

Data were collected through key informant interviews and FGDs. General Interview Guide approach (guided interview) was used to prepare checklist to ensure that all important topics are addressed [19]. Digital voice recorder was used to audio-tape discussions. Quiet places with maximum privacy were used for FGDs and interviews. Seven experienced and first-degree holder midwives familiar with the community norms were recruited for FGDs and in-depth interviews to elicit detailed responses as the issue was sensitive. Main topics covered were reasons for seeking ANC but not skilled attendance, perspectives on quality of ANC, perceptions on planning for birth and emergencies, decision making process for ANC and skilled attendance, and factors underlying high uptake of ANC services but low utilization of skilled attendance at birth. Both FGDs and IDIs were guided by an experienced person fluent in local language (Hadiyigna) and English.

### 2.6. Data Analysis

All IDIs and FGDs were transcribed and translated verbatim from local language to English by individuals fluent in both languages. Data were analyzed using Interpretative Phenomenological Analysis [20, 21]. Major themes were identified and compared across transcripts to determine similarities and differences in the views of respondents on ANC, delivery, and factors determining their decisions to seek skilled attendance at birth.

### 2.7. Quality Control

One-day intensive training was given to data collectors on study objective, informed consent, confidentiality of information, and interview techniques. The tool was pretested in adjacent kebele to ensure its validity and make data collectors familiar with it. Principal investigators and supervisors had ensured completeness and consistency of the gathered information by frequently checking data collection process.

## 3. Results

### 3.1. Demographic Characteristics of Study Participants

The young (20-29 years) accounted for 57.1 and 62.9% of the total IDIs and FGD participants. Significant proportions (35.7% and 42.4%) of IDIs and FGD participants have not received any formal education. For 4 (28.4%) and 19 (35.1%) of IDIs and FGD participants the delivery was their first experience (Tables 1 and 2).

### 3.2. Findings from Focused Group Discussions and In-Depth Interviews

When asked why they received only ANC and gave birth elsewhere other than health facility, four themes were identified from their responses (Table 3).

### 3.3. Poor Counseling during Antenatal Care Services

Even though period of ANC follow-up is considered a golden time for healthcare providers to deliver professional birth preparedness, the women were not satisfied with the care. Women could have given birth in health facilities if they had obtained classified information on when and where to give birth. Contrarily, ANC providers may not provide adequate health information on importance of skilled attendance during delivery. A 23-year-old young woman from the FGD said* “…I gave birth at home as I was not aware of dangers of home delivery. No one offered me a piece of advice on importance of facility based child birth. I have visited health facility three times for ANC, during my entire visit they checked for abnormalities and gave me some tablets to take. From my visits what I learnt was all about balanced diet, taking enough rest and taking my tablets regularly. I never remember being counseled about place of delivery. If they [the health professionals] failed to offer this information, how could poor women like me choose the right place to deliver our babies?”*

Clients have every right to get full information from providers and providers have obligation to make sure that the woman had all her questions answered and misconceptions cleared; but this is unlikely for many rural Ethiopian women. This is why some women blamed care providers for their home delivery saying “*we are pretty much concerned about our families health and we pay due attention to care providers word. We take their advice seriously, we don't even question. But they merely talk to you when they check on you. If it is your lucky day and they talk to you, it is not different from the word they have with you yesterday. Take my experience!, they were asking me the same questions every single time of my visit. I expected more but their approach made me question facility birth. If they [care provider] had advised me on this I would have given birth at health facility. But it didn't happen because of their gaps *(A mother of four from FGD 3).”

For some, ANC sessions were so boring and have inhibited them from seeking delivery care. Some clients perceived the care as unnecessary repetitive procedure as providers fail to explain what they do and why. A 37-year-old key informant said* “Why do I waste my time by visiting them? By mistake, they didn't do or tell me something different. The follow-up….it was quite a routine and I believe it is not important for a healthy woman unless she is ill. You see, we expect more from the providers, but when they fail to meet our expectations we lose courage to visit them again.” *

Some women saw ANC as a means of confirming normal pregnancy and arrange home delivery if everything is okay. Facility based childbirth would not be considered unless the provider suggested that either the baby or mother were in danger. In contexts like Ethiopia where most of rural women have no education or low (if any), such misconception is common. A 27-year-old woman from FGD said* “ANC care providers can predict any future child birth complications. So, if the provider told me my pregnancy is super fine, what is the point of giving birth in health facility? I consider facility delivery only if healthcare providers strictly told me to do so. Otherwise I don't see its importance.”*

Others attend ANC follow-up only for tablet supplements, thinking they will never get it elsewhere. For instance a teenage participant in FGD said “…*I got tablets (folic acid and iron pills) from them [providers] and it helped my baby to be healthy*.* They had told me that my pregnancy was fine and going good. This helped me decide to deliver at home in the hands of my relatives.”*

There were also women who thought ANC by itself can actually diminish risk of complications at birth. Some women are found to have a strong belief that ANC reduces the probability of developing complication. One 30-year-old key informant said* “facility based delivery is for those who don't have any follow-up. Because, they didn't know if their pregnancy is normal or not; plus they were not taking pills (Iron and Folic acid). A woman on follow-up definitely doesn't need to revisit health facility to give birth. ANC will obviously decrease her chance of getting into trouble.”*

### 3.4. Traditions and Beliefs

The psychological and social gap between care providers and community is barrier to facility based childbirth. Besides this difference between them, local understanding of disease etiology and traditional and familial influences also play key roles in deciding where to give birth. Some women described attending birth as a “routine event” and believed childbirth is a woman's “natural rite of passage”. Therefore, it is illogical to deliver in a facility, and any payment for delivery service is considered irrational. Some participants of FGD mentioned that facility based childbirth is recommended only for a woman who needs special treatment. Thus, women are encouraged to deliver themselves. One 32-year-old participant from FGD commented,* “…birth is a natural life event not a disease; therefore I don't see a reason for seeking a facility delivery and most of all spend my money unnecessarily. So, why do I expose myself to silly procedures, while I can give birth at home with a traditional birth attendant? Facility based child birth is only for weak women or those who have previous complications. In our society, a woman who delivers herself enjoys much respect.” *

The community has a long-time tradition of utilizing TBAs that had been the only delivery care givers for years, and some are still comfortable with their services. Privacy is critical issue for rural Ethiopian women, which is difficult to achieve in a facility due to lack of private labour wards or cultural insensitivity. Some women fear clinical procedures such as unfamiliar birth positions and vaginal physical examinations. A 39-year-old key informant stated,* “… for instance when my neighbor gave birth in a facility, health care providers invaded her privacy and conducted too many vaginal examinations, which was dehumanizing and uncomfortable. So how can I go to health facility knowing what my friend had experienced? I preferred to give birth at home with a traditional birth attendant as she can give me more privacy and control over the situation than care providers at health facility.” *

Recognizing the need for healthcare is vital to ensure proper healthcare seeking behavior. With this regard lack of awareness on importance of skilled attendants was observed. For some participants, assistance of skilled attendants during childbirth was viewed as compulsory only when obstetric complications arose. For women, delivery is often not perceived as an event requiring medical attention but rather a natural event and normal work. This is why women first go for home delivery and consider facility as a second option only if complications occurred. Majority of mothers who participated explained the issue using the following expression: “*we first attempt home delivery and consider facilities only if complications emerge. Though facility birth is not first choice for many women in our community, we admit its significance in cases of complicated births. But, we don't simply run to a facility for the simple and sever cases to waste our time and money*” (FGD 4).

For some, decision for accessing ANC and skilled delivery service were made differently. In many contexts women decide whether to attend or not ANC independently, but they need their husband's approval to receive facility based delivery care. This somehow indicates husband's unparalleled role in decision making process of delivery service. Obtaining approval may delay or deter facility based childbirth, mainly as these decisions are usually pursued after labour has initiated and a husband may not allow institutional delivery because of cultural or financial issues. A 24-year-old woman from the IDI said:

…*usually a woman receives antenatal care in a matter of hours and it does not as such interfere with her regular household duties. Thus, she does not need to ask for permission either her husband or any family member. But in case of delivery she has to spend a minimum of three days in a facility and could not control her domestic responsibilities like child care, tending the livestock, cleaning, and cooking. But, if gave birth at home, a woman rests in her own bed after delivery; she will not need to arrange transportation, and be comforted by her family. This is why husbands' took a lion share in the decision process of delivery location.*

### 3.5. Early Pregnancy Symptoms

In our traditional community culture of regular checkup is so poor that people visit health institutions only if they face compelling health problems. The discomfort through first trimester was a driving force for some women to attend ANC. They usually perceive physiologic pregnancy changes and discomforts as a pathological disease; they sought treatment, not ANC. These women will no longer go to health facility once the discomfort is gone. Focused group discussant and key informants also confirmed this. One key informant in her early twenties said:

*From the beginning I visited the facility as I was suffering from continues abdominal discomfort every morning [morning sickness]. On my first visit the care provider gave me an appointment to visit her again but once I got relief from my problem I didn't see the importance of repeatedly visiting her. Even she didn't stress the appointment, so how can I take it serious?*

### 3.6. Lack of Planning in Advance for Childbirth

#### 3.6.1. Financial Constraint

Local transportation is often perceived as too expensive and hard to find. In addition, cost of services had pushed participants to prefer TBAs' service. The average cost for facility based delivery was believed to be high-priced by some women. Flexibility of the payment method for TBAs was also a plus for some.

Providers may not stress relevance of planning ahead, only proposing skilled attendance for women with foreseen complications. As evidenced from the discussion, lack of planning in advance for childbirth on transportation, location of delivery, and acquiring liquid assets to pay for related costs prevented women from accessing facility delivery. A 28-year-old woman from FGD reported the following: “…*I was working the whole day normally but at night the labor came out of nowhere and took my breath. My family was disturbed and my husband went out to bring car but came all alone and told me they asked him money that he didn't had. If I was prepared economically I would have used the car and delivered in health institution, but it never happened and I gave still birth. I lost my baby*.”

#### 3.6.2. Sudden Onset of Labour

When women visit health facilities for ANC follow-up it is expected that they will get information on their approximate expected date of delivery if not the exact one. But many women were not fortunate enough to get this pertinent information. As a result for some women onset of labour could be sudden thereby prompting home delivery. A 23-year-old participant mentioned the following:* “I thought I had one more week but I was mistaken. My labour started unexpectedly and didn't gave me time to go to health institution, so I gave birth in my home with help of my neighbors. I was not aware of my exact date. Had it been according to my plan I would have given birth to my child in the health facility on the hands of the health care providers.” *

#### 3.6.3. Lack of Accessible and Reliable Transportation

Nearness to healthcare facility is a primary issue in choosing facility based childbirth. Transportation problems and difficult road conditions are linked with high costs of visits to health institutions. Cost of transportation and roads condition were mentioned as barriers to facility based childbirth. Many mention distance as a reason for women's use of TBAs compared to skilled attendants. Onset of labour is among unpredictable life experiences woman ever go through and they do not have control over what happens next. A 27-year-old woman who had labour at night from FGD explained how distance and difficulties in access led her to home delivery:* “Childbirth is an unpredictable event difficult to plan. It was at night and raining when I started labouring. My husband and his friends went out to look for a car to take me to health center but couldn't get one. Then they asked me if I can walk, but let alone in labour it was really hard for me to reach the facility during my good days [for antenatal care]. I travel considerable time to reach a facility even during normal days. So as I couldn't make it to the facility they called a traditional birth attendant and she helped me out.”*

## 4. Discussion

There has been universal agreement on importance of ANC in improving maternal and perinatal outcome; indeed it was one of four pillars of safe motherhood initiatives. ANC is a unique chance for health information dissemination on delivery, infant care, and also contraception. The service, however, requires skilled and sensitive professionals to be effective [22]. After global review of the traditional ANC, World Health Organization (WHO) implemented Focused Antenatal Care (FANC) which focuses on quality of services given rather than number of visits [2]. In addition to promotion of facility delivery, FANC gives opportunities to deal with many conditions directly or indirectly related to pregnancy such as malaria, TB, and STIs including HIV [23]. Why then significant number of women continue to give birth at home despite receiving ANC? This was central question of this research and four themes emerged: poor counseling during ANC, traditions and beliefs, early pregnancy symptoms, lack of birth plan, and sudden onset of labour as main reasons.

Women satisfied with quality of ANC they had received have increased odds of using skilled attendance at birth compared with those with low level of satisfaction [24]. This solid connection between quality of ANC and uptake of skilled delivery service may explain the contradicting pattern in high use of ANC and low use of facility based delivery [25]. Thus, ANC has to be individualized and respectful at every contact and has to be provided by professionals with good interpersonal and clinical skills within a well-functioning health system. Healthcare professionals are expected to discuss danger signs of pregnancy, birth plans and healthy eating and perform maternal and fetal assessments [23]. It is known that danger signs are critical indicators of pregnant mother's health status; lack of knowledge on danger signs would lead to deferment to seek care. This study revealed that a significant number participants were not told anything about danger signs of pregnancy and importance of facility based childbirth. A review of studies conducted in Sub-Saharan Africa also revealed that many women in Ethiopia have high unmet need for information on pregnancy complications, and only 29% of women were advised on institutional delivery during their antenatal follow-up. This lack of information may hinder women's ability to enjoy facility based delivery care [26].

Participants of this study believed that pregnancy is not a disease; hence there is no need to visit health facility and they preferred TBAs at childbirth; moreover healthcare providers told them that their pregnancy has no major problem. Such discussion has been outdated by evidence based findings saying that every pregnancy carries its own risks. However, their TBA preference at childbirth could also be due to cost, perceptions on care providers' skills, tradition, and previous experience with TBA [27–29].

Discussion on birth plan and place of delivery is among core activities during ANC visit. Though professionals might have discussed it, participants mentioned they were unable to visit facility due to financial constraints proving that there was no birth preparedness and complication readiness plan. Several studies also revealed that costs are among deterrents to facility based delivery [30, 31].

Women arrive at a decision about facility delivery based on options, information, understanding, and previous experience. Several studies showed that previous negative experiences such as disrespect and abusive treatments at health facilities are deterring factors to facility based childbirth [28, 29, 32–36]. Following this disturbing research outcome WHO issued a statement to prevent and eliminate disrespect and abuse at health facility during delivery care [37]. Likewise, study participants mentioned that they either heard or had negative experiences at health facility which enforced them to deliver at home. This shows that acceptability and quality of delivery care are very poor, violating woman's fundamental human rights. Therefore, the health system has to be accountable for such maltreatment and is expected to support as well as train care providers to be compassionate, respectful, and caring.

Since 1990s Ethiopian government has expanded health infrastructures and work force throughout the country. However, access and reliable transportation remain unfinished agenda. In this study, participants mentioned lack of transportation and distance as constraints to deliver at health facility. It could be related to poor road infrastructures, weak transportation network, and residential houses being distant from each other especially in rural areas. Numerous studies had proved that the closer the distance is, the more likely the women will utilize facility for delivery [31, 38, 39].

In this study participants were not adequately told about ANC service and their visits were left in vain as long as they did not deliver at facility. A study conducted in Northern Ethiopia showed that maternal education is a determinant factor for facility delivery among ANC attendees [35]. It is known that literacy rate is very low in Ethiopia [40] especially among mothers; given their low schooling, ANC service delivery has to be to their level of understanding, because health facility is the only place pregnant mothers could access targeted information and services. On the other hand, most women might attend health posts (closest health facility in the nation) where health extension workers work. But health extension training program is of short duration and low quality and their role in maternal health remains controversial [27]. Hence, they are less likely to provide comprehensive ANC service.

Ethiopia achieved Millennium Development Goal 4 which was reduction of child mortality; however neonatal mortality remains high [16, 41]. Several studies showed that one-third of neonatal mortality occurs during intrapartum period; moreover countries with highest neonatal mortality are countries whereby more than half of deliveries occur outside health facility [42, 43]. This shows how quality ANC and skilled delivery care are crucial in averting not only maternal but also neonatal death. This study found out that ANC use does not lead to facility delivery although most studies found it to be strong predictor. This variation could be explained by variation in design nature.

## 5. Conclusion

Although Ethiopian government has implemented high impact interventions to improve maternal health services, it does not go beyond access as this study revealed. On the basis of our findings, the gap between ANC use and facility delivery is related to quality of ANC and other individual and health system factors. Hence, it calls for policy makers and ANC program planners to give due emphasis on quality of ANC service and focus on shaping pregnant mothers attitude by reinforcing individual messages and responsibility so as to make the interaction between ANC providers and the clients meaningful.

Our results provide a starting point to explore reasons for home delivery despite ANC utilization; further studies are recommended to explore the view of health professionals and policy makers.

## Figures and Tables

**Table 1:** Sociodemographic characteristics of in-depth interview participants, Hadiya zone, Ethiopia, 2016.

**Characteristics**	**14**	**Number**	**Percentages**
**Age **	20-29	8	57.1
	≥30	6	42.8
**Education **	No education	5	35.7
	Primary	4	28.5
	Secondary	3	21.4
	Above secondary	2	14.2
**Number of children**	1	4	28.4
	2-3	7	50
	≥4	3	21.6

**Table 2:** Sociodemographic characteristics of focused group discussion participants, Hadiya zone, Ethiopia, 2016.

**Characteristics**	**54**	**Number**	**Percentages**
**Age **	20-29	34	62.9
	≥30	20	37.2
**Education **	No education	23	42.4
	Primary	14	25.9
	Secondary	11	20.5
	Above secondary	6	11.3
**Number of children**	1	19	35.1
	2-3	23	42.6
	≥4	12	22.3

**Table 3:** Categories of themes and subthemes.

**No.**	**Theme**	**Subthemes**
1	Provider factors	Poor counseling during antenatal care services
2	Traditional factors	Traditions and beliefs
3	Personal factor	Early pregnancy symptoms
		Lack of planning in advance for childbirth
		(i) Financial constraint
		(ii) Sudden onset of labour
		(iii) Lack of accessible and reliable transportation

## Abbreviations

ANC: Antenatal care
FANC: Focused Antenatal Care
FGDs: Focused group discussions
HC: Health center
HP: Health post
IDIs: In-depth interviews
TBAs: Traditional birth attendants
WHO: World Health Organization.
